# Bioinformatic Analysis Reveals GSG2 as a Potential Target for Breast Cancer Therapy

**DOI:** 10.1515/biol-2019-0078

**Published:** 2019-12-31

**Authors:** Zheng Ye, Zhaoyu Zhang, Lijiao Fang, Daiquan Tian, Xin Liu

**Affiliations:** 1 Department of Biochemistry and Molecular Biology, 22 Qixiangtai Road, Tianjin Medical University, Tianjin 300070, China; 2 Tianjin Key Laboratory of Medical Epigenetics; Key Laboratory of Breast Cancer Prevention and Therapy (Ministry of Education); Department of Biochemistry and Molecular Biology, Tianjin Medical University, Tianjin 300070, China; 3 Tianjin Chest Hospital, Tianjin 300222, China

**Keywords:** GSG2, Breast cancer, CNV

## Abstract

**Objective:**

To explore the potential role of GSG2 in breast cancer progression.

**Methods:**

The mRNA expression, DNA copy number and clinical data used in this study were obtained from the TCGA data portal. The copy number variations (CNVs) thresholds were determined according to the set of discrete copy number calls provided by Genomic Identification of Significant Targets in Cancer (GISTIC).

**Results:**

The mRNA expression level of GSG2 in 112 breast cancer tissues was much higher than that in adjacent normal tissues. GSG2 was significantly upregulated in stage II compared with stage I, and there was no differential expression of GSG2 between tumors with or without metastasis. Heterozygous deletion occupied 57.1% of CNVs for GSG2 gene in breast cancer samples. Patients with higher GSG2 expression tended to suffer from poorer prognosis.

**Conclusion:**

Our profiling analysis indicated the overexpression of GSG2 might play an important role in breast cancer development, suggesting that GSG2 could be a new target for breast cancer treatment, making GSG2 inhibitors becoming potential drugs for breast cancer therapy.

## 1 Introduction

According to the Global Cancer statistics, there were an estimated 18.1 million new cancer cases and 9.6 million cancer deaths in 2018 [1]. Breast cancer is the second commonly diagnosed cancer and the second leading cause of cancer death [1], which makes it essential to study the mechanism of breast cancer progression and to find new targets for breast cancer treatment.

The mRNA of Haspin was first discovered in male germ cells of mice, therefore, the gene and protein were given the names GSG2 (germ cell-specific gene 2) and HASPIN (haploid cell-specific protein kinase), respectively [2]. Haspin localizes in nucleus in interphase cells [2, 3], and is predominantly associated with chromosomes, especially centrosomes in mitosis [4, 5]. Despite the absence of some of the highly conserved motifs found in canonical eukaryotic protein kinases, mammalian HASPIN proteins have been proved definitively to have serine/threonine kinase activity, and its only substrate is histone H3 [4, 6, 7]. Haspin phosphorylates histone H3 during mitosis and plays an important role in regulating chromosome behavior during cell division [8]. Haspin depletion results in a defect in chromosome congression and a delay in exit from mitosis [9, 10].

In our study, we found that the transcription of GSG2 gene is upregulated in breast cancer tissues and its expression changes across different PAM50 subtype breast cancer samples. GSG2 mRNA expression is significantly upregulated in stage II breast cancer compared with stage I. Furthermore, CNV analysis indicated that CNV is not the main reason for the upregulation of GSG2 in breast cancer. Finally, we showed that patients with higher GSG2 mRNA expression tend to suffer from poorer prognoses. Our study makes GSG2 gene a potential new target for breast cancer treatment.

## 2 Methods

### 2.1 Data source and curation

The mRNA expression, DNA copy number and clinical data used in this research were obtained from TCGA data portal (http://portal.gdc.cancer.gov/). The 112 paired breast cancer and adjacent tissue samples were used to analyze the GSG2 gene differential expression. P value was determined by paired t-test.

A total of 1080 tumor samples were used to analyze the mRNA levels of GSG2 among four stages and four PAM50 subtypes according to clinical data. P values between any two groups in stage analysis or PAM50 subtype analysis were determined by Mann-Whitney U test.

To test the robustness, 60% of the original samples were randomly picked up to test the expression difference and repeated 100 times. Sample function in the R package “base” was used to randomly selected samples. R package “sampling” was used to selected samples by stratified sampling.

To validate the results of the TCGA data, the mRNA expression data; CNV data; and clinical information of METABRIC dataset (http://www.cbioportal.org/datasets) which contains 1904 tumor samples were also downloaded and analyzed.

### 2.2 CNV and expression relationship

To determine the copy number status of GSG2, Genomic Identification of Significant Targets in Cancer (GISTIC) algorithms (http://software.broadinstitute.org/cancer/cga/gistic) was used to analyze the segmentation files downloaded from TCGA. The CNV thresholds were determined according to the set of discrete copy number calls provided by GISTIC: homozygous deletion (-2), heterozygous deletion (-1), diploid (0), gain (1) and amplification (2). The Mann-Whitney U test was used to determine whether the mRNA levels differences were significant between any two CNV statuses.

### 2.3 Survival analysis

To perform Kaplan-Meier survival analysis, R package “survival” was used to analyze TCGA BRCA clinical data and expression data in tumor samples. The low expression was defined by expression levels lower than the median (n = 539) and high expression was defined by expression levels higher than the median (n = 540). The log-rank test was used to compare the difference of survival curves. P value < 0.05 was considered statistically significant.

All analyses were performed using R 3.5.1.

## 3 Results

### 3.1 The mRNA expression levels of GSG2 are higher in breast cancer tissues compared to their normal counterparts

**Figure 1:**
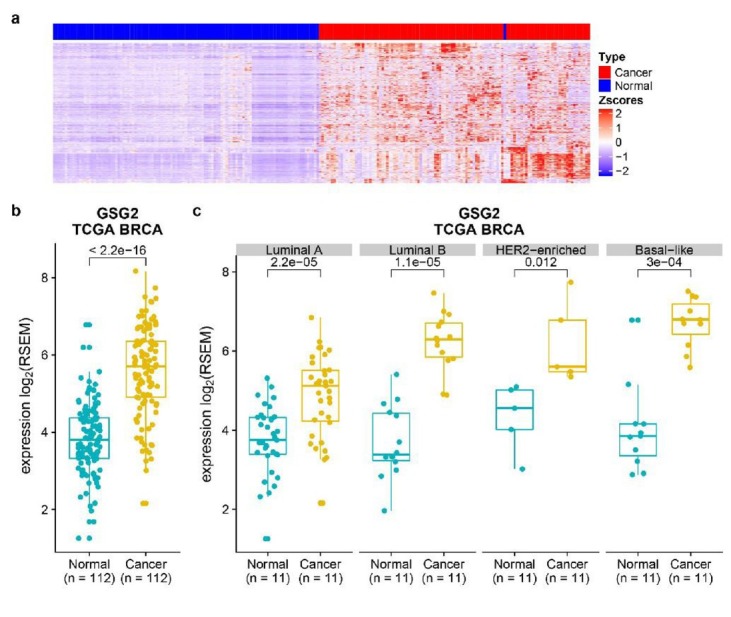
The mRNA expression levels of GSG2 are higher in breast cancer tissues compared to normal counterparts. a. Expression levels of 17805 genes in breast cancer and adjacent tissue pairs (n = 112) were analyzed and expression heat map was shown. Statistical analysis was performed using paired t-test (p < 0.001). b. Data for the GSG2 mRNA expression levels in breast cancer tumor and normal tissue pairs were calculated. Box plots showed the differential expression of GSG2 between breast cancer (n = 112) and normal (n = 112) samples. Statistical analysis was performed using paired t-test (p < 0.001). c. The mRNA expression levels of GSG2 were all higher in tumor tissues than normal tissues according to PAM50 intrinsic subtyping. Box plots showed the differential expression of GSG2 between cancer and normal samples in luminal A (n = 35 pairs), luminal B (n = 14 pairs), HER2-enriched (n = 5 pairs) and basal-like (n = 11 pairs) subtypes, respectively. Statistical analysis were performed using paired t-test (p < 0.001 in luminal A, luminal B and basal-like tumor samples, p = 0.012 in HER2-enriched tumor samples).

It has been reported that many mitotic players are often upregulated in tumor samples due to an elevated mitotic index in tumor cells [11, 12]. Our previous works focused on proteins or factors involved in mitosis and their roles in tumorigenesis and tumor development. To examine the differential expression genes in breast cancer, we downloaded the TCGA BRCA data set, and calculated the expression difference of 17805 genes between 112 tumor-normal paired samples. The results showed that there are 877 genes upregulated in breast cancer samples (P value < 0.001) (Figure 1a). GSG2 protein is essential for proper chromosome congression during mitosis. To investigate the potential function of GSG2 in breast cancer, we focused on its mRNA expression level in breast cancer tissues. The analysis revealed that the mRNA expression level of GSG2 in breast cancer tissues is much higher than that in adjacent normal tissues (Figure 1b).

**Figure S1:**
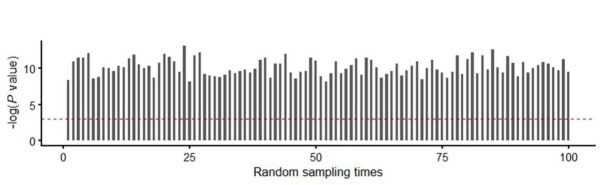
Sixty percent of the original samples were randomly picked up to test the expression difference and repeated 100 times. Bar plots showed the –log (P values) of 100 time statistical analysis. P values were determined by paired t-test. The value of –log (0.001) appear as red line.

To test the robustness, 60% of the original samples were randomly picked up to test the expression difference and repeated 100 times. The results confirmed the differential expression of GSG2 between breast cancer tissues and adjacent tissues (Fig. S1).

**Figure 2:**
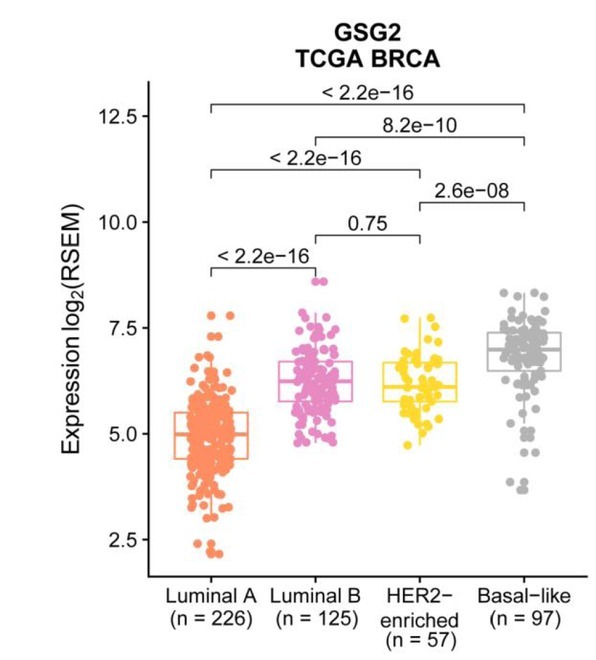
The expression levels of GSG2 changes across different PAM50 subtype breast cancer samples. Box plots showed the differential expression of GSG2 in luminal A (n = 226), luminal B (n = 125), HER2-enriched (n = 57) and Basal-like (n = 97) tumor samples. P values were determined by Mann-Whitney U test.

**Figure 3:**
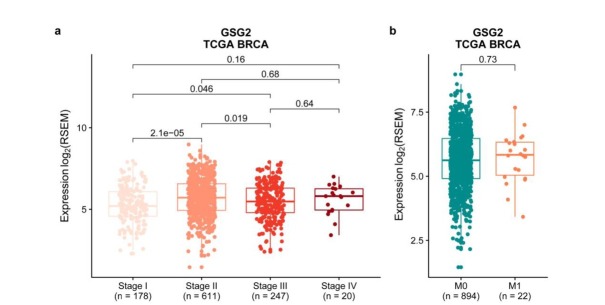
The expression level of GSG2 is higher in stage II tumor samples and is not associated with metastasis. a. Box plots showed the differential expression of GSG2 across tumor samples in stage I (n = 178), stage II (n = 611), stage III (n = 247) and stage IV (n = 20). b. Box plots showed the expression of GSG2 in metastatic tumor samples (M1, n = 22) and non-metastatic tumor samples (M0, n = 894). Statistical analysis was performed using the Mann-Whitney U test.

**Figure 4:**
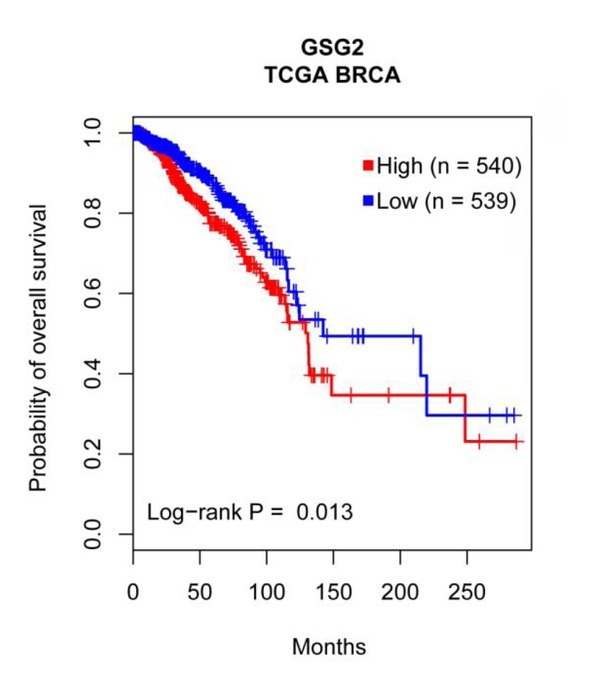
GSG2 genomic copy number variations (CNVs) mainly centered on heterozygous deletion. a. Data for the GSG2 CNVs were downloaded from the TCGA dataset and analyzed by GISTIC. Bar plot showed the distribution of GSG2 CNVs across tumor samples and heatmaps showed percentage of GSG2 CNVs in different stages. The percentages were calculated by row. b. Different CNVs statuses of GSG2 were plotted against the corresponding mRNA expressions of the gene (Homozygous Deletion: n = 6, Heterozygous Deletion: n = 620, Diploid: n = 383, Gain: n = 68, and Amplification: n = 3). Statistical analysis was performed using the Mann-Whitney U test.

**Figure 5:**
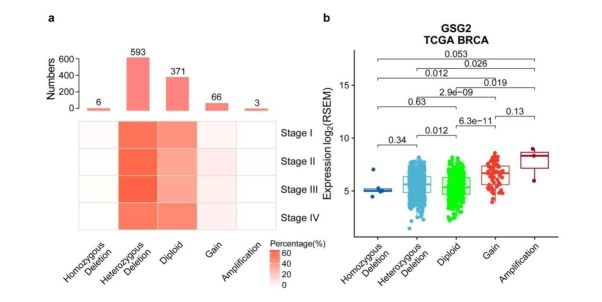
Kaplan-Meier plot of overall survival associated with the mRNA expression level of GSG2 in TCGA BRCA. The x axis was the overall survival (OS) month, and the y axis represented the survival rate. Statistical analysis was performed using log-rank test.

**Figure S2:**
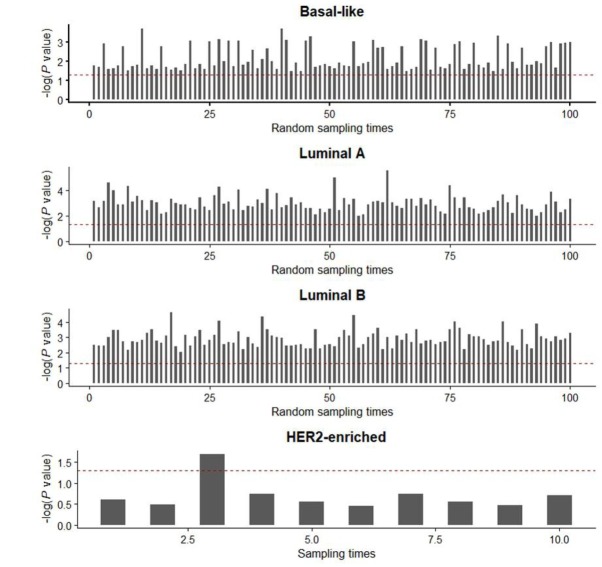
Sixty percent of the original samples were randomly picked up to test the expression difference and repeated. Bar plots showed the –log (P values) of 100 times statistical analysis for luminal A, luminal B, and basal-like subtypes and the –log (P values) of 10 times statistical analysis for HER2-enriched. P values were determined by paired t-test. The value of –log (0.05) appear as red line.

According to breast cancer PAM50 intrinsic subtyping criteria, we divided these breast cancer samples into four types (luminal A, luminal B, Her2-enriched, and basal-like breast cancer), and analyzed the mRNA expression of GSG2 in tumor and adjacent normal tissues. The results showed that the mRNA levels of GSG2 in these four subtypes of breast cancer samples are all significantly higher than that in the corresponding adjacent samples (Figure 1c and Fig. S2).

### 3.2 The expression levels of GSG2 changes across different PAM50 subtype breast cancer samples

**Figure S3:**
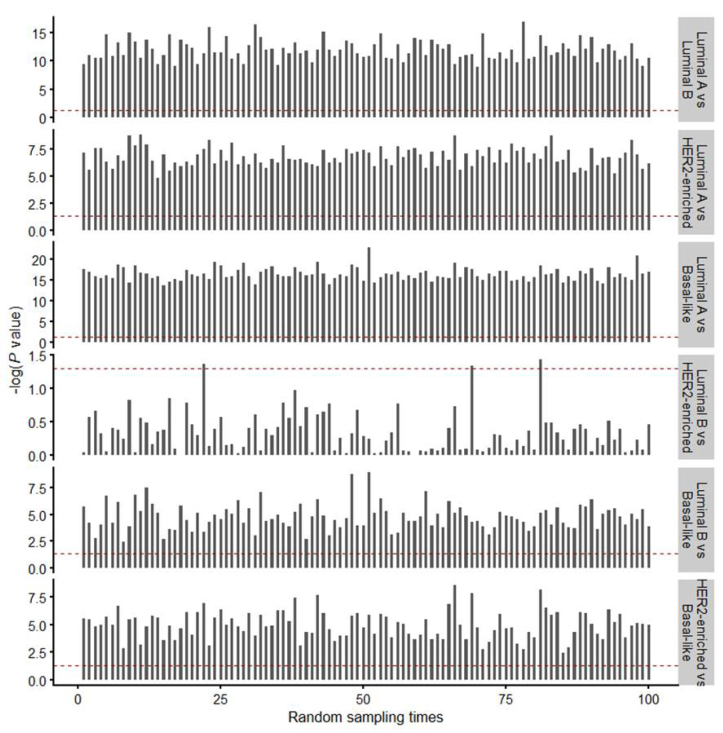
Differential expression of GSG2 from 60% of original PAM50 subtype cancer samples. Bar plots showed the –log (P values) of 100 times statistical analysis for any two PAM50 subtype groups. P values were determined by paired t-test. The value of –log (0.01) appear as red line.

Furthermore, we analyzed the expression levels of GSG2 in different PAM50 subtype breast cancers. It turns out that GSG2 expression in luminal A breast cancers is the lowest and its expression in basal-like breast cancer is the highest in these four subtypes of breast cancer samples (Figure 2 and Fig. S3). Analysis using METABRIC dataset showed consistent results with TCGA dataset (Fig. S5).

**Figure S4:**
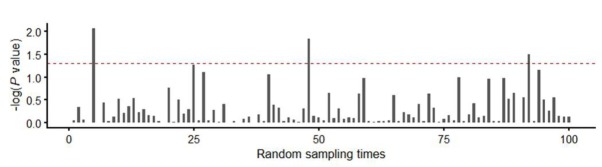
Differential expression of GSG2 from 60% of metastatic and non-metastatic samples. Bar plots showed the –log (P values) of 100 times statistical analysis. P values were determined by t-test. The value of –log (0.05) appear as red line.

**Figure S5:**
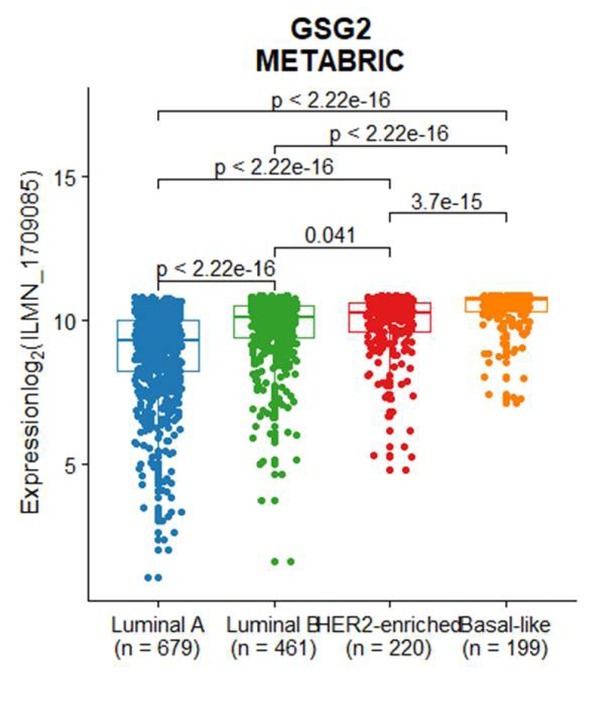
The expression levels of GSG2 changes across different PAM50 subtype breast cancer samples. Box plots showed the differential expression of GSG2 in luminal A (n = 679), luminal B (n = 461), HER2-enriched (n = 220) and Basal-like (n = 199) tumor samples. P values were determined by Mann-Whitney U test.

### 3.3 The expression level of GSG2 is higher in stage II tumor samples and is not associated with metastasis

**Figure S6:**
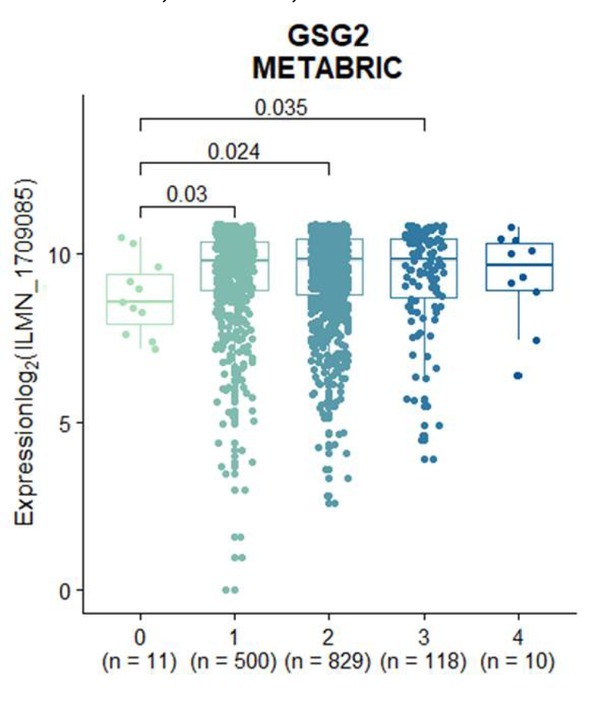
The expression level of GSG2 is higher in early tumor samples. Box plots showed the differential expression of GSG2 across tumor samples in stage 0 (n = 11), stage 1 (n = 500), stage 2 (n = 829), stage 3 (n = 118) and stage 4 (n = 10).

To further study the probable role of GSG2 in the progression of breast cancer, we analyzed the difference of GSG2 expression in 1,056 breast cancer samples classified according to tumor stages, and found that GSG2 is significantly up-regulated in stage II compared with stage I (Figure 3a), suggesting that the increase of GSG2 expression may play an important role in the development from stage I breast cancer to stage II. Moreover, there was no difference in the expression of GSG2 between tumors with or without metastasis (Figure 3b and Fig. S4), indicating that GSG2 may not be involved in breast cancer metastasis. Results from the METABRIC dataset are consistent with that from the TCGA dataset as well (Fig. S6).

### 3.4 CNVs are not the main reason for the upregulation of GSG2 in breast cancers

DNA copy number variations (CNVs) can result in the upregulation of oncogenes and downregulation of tumor suppressors in human cancers [13], and CNV-derived gene dysregulation is common in various cancers [14, 15]. Therefore, we hypothesized that the overexpression of GSG2 might originate from increased copy number of this gene. To test this, we calculated the copy number variation distribution of the GSG2 gene in 1,080 breast cancer samples. The results showed that there are 593 breast cancer samples (57.1%) containing heterozygous deletion of the GSG2 gene, however, there is a very low frequency of GSG2 gene amplification (< 1%) and GSG2 gene gain (< 7%) in breast cancer samples (Figure 4a). We also analyzed the variation of the copy number of the gene in relation to the clinical stage and found that the three tumor samples with GSG2 gene amplification were all in stage II, and GSG2 gene gain happened most frequently in stage II among the four stages (Figure 4a).

**Figure S7:**
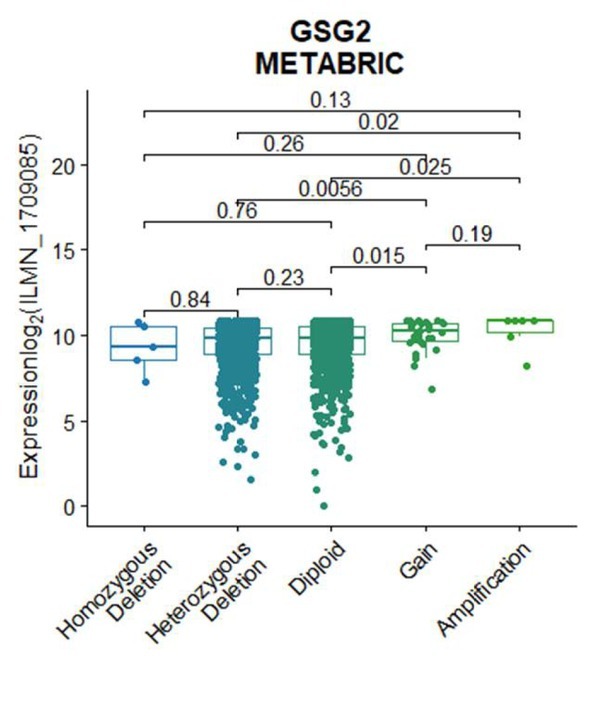
GSG2 genomic copy number variations (CNVs) mainly centered on heterozygous deletion. Different CNVs statuses of GSG2 were plotted against the corresponding mRNA expressions of the gene (Homozygous Deletion: n = 5, Heterozygous Deletion: n = 929, Diploid: n = 934, Gain: n = 30, and Amplification: n = 6). Statistical analysis was performed using the Mann-Whitney U test.

We continue to explore the relationship between copy number variability and expression level of GSG2. The analysis showed that GSG2 expression in tumors with the GSG2 gene gain and GSG2 gene amplification is evidently higher than diploid (Figure 4b and Fig. S7). However, the GSG2 expression in tumor samples with homozygous deletion of the gene (the most common pattern of GSG2 genomic copy number variations in breast cancer tissues) is not significantly lower than that in tumor tissues with diploid (Figure 4b and Fig. S7). These results indicated that CNVs are not the main reason for the upregulation of GSG2 in breast cancers, suggesting that there are important transcription factors and epigenetic factors upregulating GSG2.

### 3.5 Higher GSG2 expression is associated with poorer prognosis of breast cancer patients

**Figure S8:**
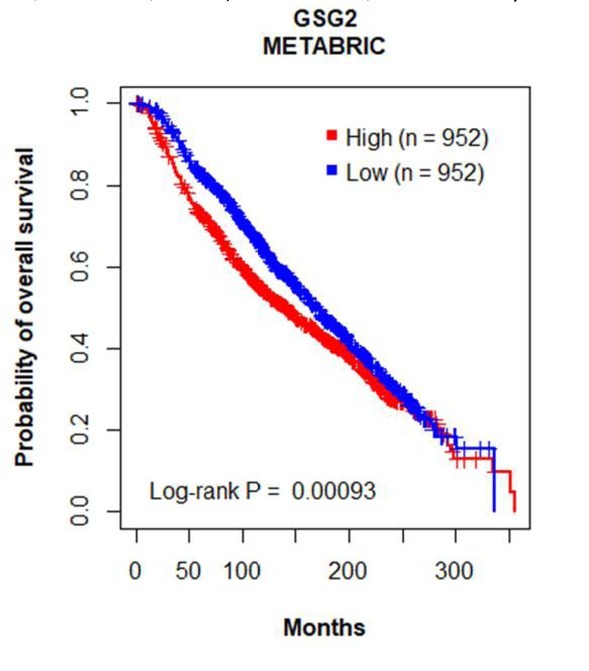
Kaplan-Meier plot of overall survival associated with the mRNA expression level of GSG2 in METABRIC dataset. The x axis was the overall survival (OS) month, and the y axis represented the survival rate. Statistical analysis was performed using log-rank test.

Furthermore, we employed the TCGA and the METABRIC dataset for survival analysis to reveal the potential prognostic value underlying the GSG2 overexpression in breast cancer tissues. The results indicated that patients with higher GSG2 expression tend to suffer from poorer prognoses (Figure 5 and Fig S8).

## 4 Discussion

GSG2 has been proved to be a serine/threonine kinase that phosphorylates histone 3 during mitosis and plays an important role in regulating chromosome behavior during cell division [3, 6, 7]. Because of its essential activities in mitosis, GSG2 inhibitors have been developed as potential anti-cancer drugs recently [16, 17, 18, 19]. Lili Han et al found that haspin inhibitor CHR-6494 inhibited the viability of several melanoma cell lines [20]. And Jong-Eun Kim et al investigated a natural compound, coumestrol, which exhibits broad anti-cancer effects against skin melanoma, lung cancer and colon cancer cell growth due to the direct targeting of GSG2 [21].

In our present study, we found that transcription of the GSG2 gene is upregulated in breast cancer tissues, and the differential expression occurs in four PAM50 subtypes breast cancer samples. GSG2 mRNA expression is significantly upregulated in stage II breast cancer compared with stage I, indicating that the increase in GSG2 expression may play an important role in the development of breast cancer. To investigate the reason to give rise to its overexpression, we analyzed DNA copy number variations of the *GSG2* gene and found that heterozygous deletion occupies 57.1% of 1,080 breast cancer samples. Further exploration showed that GSG2 expression in tumors with GSG2 gene gain and gene amplification is evidently higher than diploid. However, the GSG2 expression in tumor samples with homozygous deletion of the gene is not significantly lower than that in tumor tissues with diploid. Although CNV-derived gene dysregulation is common in human cancers [14, 15], CNVs are not the main reason for the upregulation of GSG2 in breast cancers based on our analysis, suggesting that there are important transcription factors and epigenetic factors responsible for upregulating GSG2. Finally, TCGA data showed that patients with higher GSG2 mRNA expression tend to suffer from poorer prognosis.

Our findings suggest that GSG2 could be a new target for breast cancer treatment, making GSG2 inhibitors potential drugs for breast cancer therapy.
